# ZnS/CoS@C Derived from ZIF-8/67 Rhombohedral Dodecahedron Dispersed on Graphene as High-Performance Anode for Sodium-Ion Batteries

**DOI:** 10.3390/molecules28196914

**Published:** 2023-10-03

**Authors:** Miao Jia, Wenfeng Chen, Yilin He, Yutong Liu, Mengqiu Jia

**Affiliations:** 1 College of Chemistry and Materials Engineering, Beijing Technology and Business University, Beijing 100048, China; m13310406896@163.com (Y.H.); 13263316552@163.com (Y.L.); 2 Beijing Key Laboratory of Electrochemical Process and Technology for Materials, Beijing University of Chemical Technology, Beijing 100029, China; 15155059081@163.com (W.C.); jiamq@mail.buct.edu.cn (M.J.)

**Keywords:** metal sulfides, metal organic frameworks, graphene, sodium-ion battery, anode

## Abstract

Metal sulfides are highly promising anode materials for sodium-ion batteries due to their high theoretical capacity and ease of designing morphology and structure. In this study, a metal–organic framework (ZIF-8/67 dodecahedron) was used as a precursor due to its large specific surface area, adjustable pore structure, morphology, composition, and multiple active sites in electrochemical reactions. The ZIF-8/67/GO was synthesized using a water bath method by introducing graphene; the dispersibility of ZIF-8/67 was improved, the conductivity increased, and the volume expansion phenomenon that occurs during the electrochemical deintercalation of sodium was prevented. Furthermore, vulcanization was carried out to obtain ZnS/CoS@C/rGO composite materials, which were tested for their electrochemical properties. The results showed that the ZnS/CoS@C/rGO composite was successfully synthesized, with dodecahedrons dispersed in large graphene layers. It maintained a capacity of 414.8 mAh g^−1^ after cycling at a current density of 200 mA g^−1^ for 70 times, exhibiting stable rate performance with a reversible capacity of 308.0 mAh g^−1^ at a high current of 2 A g^−1^. The excellent rate performance of the composite is attributed to its partial pseudocapacitive contribution. The calculation of the diffusion coefficient of Na^+^ indicates that the rapid sodium ion migration rate of this composite material is also one of the reasons for its excellent performance. This study highlights the broad application prospects of metal–organic framework-derived metal sulfides as anode materials for sodium-ion batteries.

## 1. Introduction

The primary objective for addressing climate change is to focus on energy conservation, emission reduction, and low carbon development. This can be achieved by prioritizing the development of new clean energy options, transforming the energy structure and minimizing the use of fossil fuels. Lithium-ion batteries are highly desirable due to their high potential and lack of memory effect. However, the low reserves of lithium in the earth’s crust and the over-exploitation have made large-scale energy storage difficult [1,2,3]. Sodium metal and lithium metal share similar physical and chemical properties, making the theories developed for lithium-ion batteries applicable to sodium-ion batteries. Additionally, sodium reserves are abundant, making sodium-ion batteries a promising substitute for lithium-ion batteries. Nevertheless, the relatively large radius of Na ions limits their application, making it essential to develop new anodes with high capacity and a long cycle life [4,5,6].

Metal sulfides have a long cycle life and high power density, making them a popular choice for various energy devices. However, transition metal sulfides have some drawbacks, including poor electrical conductivity, considerable volume changes during electrochemical cycling of Na-ion batteries, and low initial coulombic efficiency (ICE) [7,8]. To address these issues, researchers have focused on improving the electrochemical performance of metal sulfides in electrode materials by regulating the nanoscale structure of the material. This has resulted in the preparation of 1D nanowires, 2D nanosheets, 3D nanospheres, and hollow nanocubes, among other structures [9,10,11,12]. Another effective method is to compound materials, which involves combining two or more materials to complement and compound their performance, thereby improving the electrochemical properties of the materials [13]. For example, Li et al. [14] decorated SnS nanosheets on N-doped carbon materials, which increased the interlayer spacing of the material, allowing it to better adapt to volume changes during discharge and charging processes. Yin et al. [15] used polyethylene pyrrolidone (PVP) and carbon nitride-derived materials for coating to improve the poor electronic conductivity of SnS and alleviate its large volume expansion, resulting in high electrochemical performance. Carbon coating successfully enhanced the electron and ion transfer ability of the composite material and adapted to volume expansion, significantly improving the sodium storage performance for SnS electrodes. In addition, metal sulfide composite materials, such as FeS [16], CoS [17], ZnS [18], NiS_2_ [19], CuS [20], etc., have been studied to some extent. Composite materials show great potential for application in sodium-ion batteries, reducing the impact of poor rate performance and cycle life caused by poor conductivity and relatively serious volume changes during the cycle.

Metal–organic framework compounds (MOFs) have many advantages, including a large specific surface area, adjustable pore structure, morphology, and composition, and numerous electrochemical reaction active sites. MOFs made with different central metal atoms and organic ligands have varying ionic radii, coordination numbers, and electronegativity of the central atom, leading to different properties and functions [21,22,23]. In particular, MOFs are useful as anode materials for sodium-ion batteries due to their stable skeleton structure, which allows for the transmission of large sodium ions while reducing the impact on irreversible capacity. Zeolite imidazolate framework materials (ZIFs) are a type of MOF with a large specific surface area. ZIF-67 and ZIF-8 are symmetrical cubic crystal materials composed of metal ions (Co^2+^, Zn^2+^) and organic compounds (2-methylimidazole) that are highly stable and widely used in gas adsorption, molecular separation, electrochemical catalysis, and other fields [24,25]. Wang et al. [26] synthesized cobalt sulfide multi-shell nanoboxes using the complex anion conversion and exchange process of MOFs, which have enhanced sodium storage performance and can maintain a high specific capacity of 438 mAh g^−1^ after 100 cycles at a current density of 500 mA g^−1^. Li et al. [27] prepared bimetallic sulfide composite materials with coaxial carbon coating (WS_2_/ZnS heterojunction) using a simple one-step method, which significantly improved ion and electron diffusion kinetics. The uniform carbon protective layer around the surface of the composite material ensured excellent structural stability. Therefore, metal sulfides derived in situ with metal–organic frameworks are expected to possess excellent performance as anode materials for sodium-ion batteries.

Here, we used ZIF-8/67 as a precursor, compounded with graphene, and further synthesized ZnS/CoS@C/rGO composites by one-step sintering and vulcanization. After combining with graphene, the dispersion of ZIF materials further enhances the specific surface area, fully leveraging the sodium storage advantage of composite materials. On the other hand, the introduction of graphene also enhances the conductivity of composite material, so it has excellent performance in the electrochemical performance test, which maintained a value of 414.8 mAh g^−1^ after being cycled at a current density of 200 mA g^−1^ for 70 times. At the same time, it has excellent rate performance with a capacity of 308.0 mAh g^−1^ at 2 A g^−1^; in addition, it also exhibits ultra-long cycle life at a high current (374.2 mAh g^−1^ after 500 cycles at 1 A g^−1^). The pseudocapacitive calculation results show that the excellent performance of the composite material is derived from part of its pseudocapacitive contribution.

## 2. Results and Discussion

**Scheme 1:**
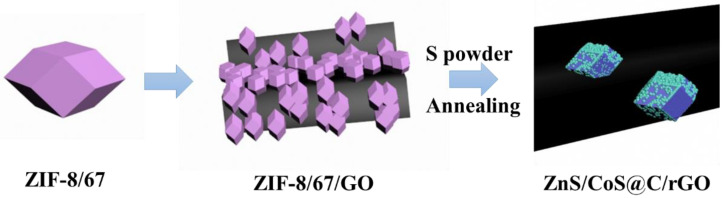
Schematic diagram of the preparation process of ZnS/CoS@C/rGO composites.

The process of creating ZnS/CoS@C/rGO composite material is detailed in Scheme 1. The first step involved preparing ZIF-8/67 using dimethyl imidazole as the precursor, followed by creating composite graphene using a water bath. The final product, ZnS/CoS@C/rGO, was obtained by sintering with sulfur powder in a tubular furnace.

**Figure 1:**
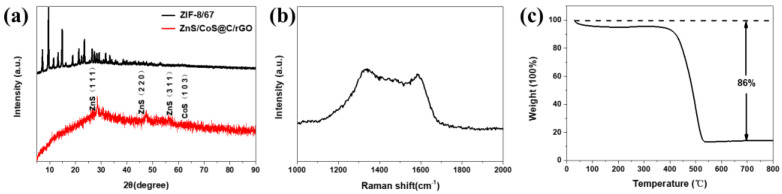
(**a**) XRD patterns of ZIF-8/67 and ZnS/CoS@C/rGO, (**b**) Raman spectrum; (**c**) TG curve of ZnS/CoS@C/rGO.

Figure 1a displays the XRD patterns of ZIF-8/67 and ZnS/CoS@C/rGO. The diffraction peaks at 28.6, 47.5, 56.3, and 62.7 represent the (1 1 1), (2 2 0), and (3 1 1) crystal planes of ZnS (JCPDS No. 05-0566) and the (1 0 3) crystal plane of CoS (JCPDS No. 01-1279), respectively. This confirms that the ZIF-8/67 precursor was successfully transformed into ZnS/CoS@C/rGO composite material through vulcanization after being compounded with graphene. Figure 1b displays the Raman spectrum of the ZnS/CoS@C/rGO composites used to characterize the degree of graphitization of carbon materials. The two main peaks at ~1334.08 and ~1581.9 cm^−1^ represent the disordered bands (D) and graphite bands (G), respectively. The intensity ratio of the two peaks (I_D_/I_G_) is 1.053, indicating that the composite material has more defects and is suitable for sodium ion storage. Figure 1c displays the thermogravimetric test of ZnS/CoS@C/rGO composites. After heating the composite material to 800 °C, the weight loss ratio of the composite material is 86%. According to previous research [28,29], the remaining products of this type of composite material after heating in air are ZnO and Co_3_O_4_, so the equation can be written as follows: 6ZnS + 6CoS + 19O_2_ = 6ZnO + 2Co_3_O_4_ + 12SO_2_. Therefore, in addition to the lost carbon, the weight loss rate also includes the volatile SO_2_. Based on this, the carbon, ZnS, and CoS contents of the composite material can be calculated as 83.7%, 8.4%, 7.9%, respectively.

**Figure 2:**
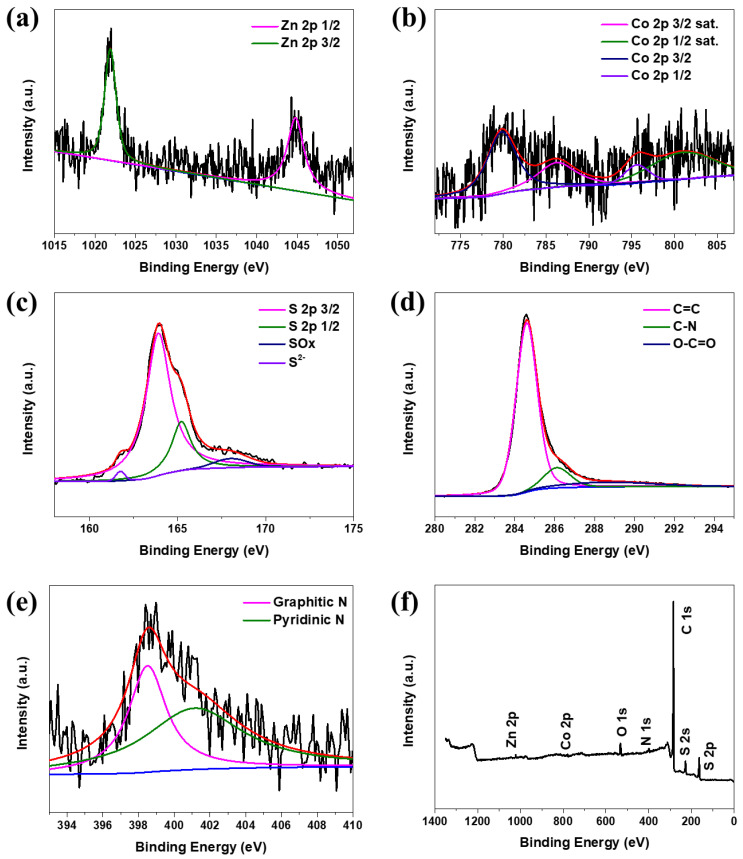
XPS spectrum of ZnS/CoS@C/rGO composite, (**a**) Zn 2p, (**b**) Co 2p, (**c**) S 2p, (**d**) C 1s, (**e**) N 1s, (**f**) survey.

The composite materials were analyzed using XPS spectroscopy analysis to determine their surface chemical valence and elemental composition. Figure 2a shows two main peaks at 1021 eV and 1044 eV, representing the Zn2p 3/2 and the Zn2p 1/2, indicating the existence of Zn^2+^ states. The XPS spectrum of the Co element is presented in Figure 2b, where the binding energy peaks of Co2p 3/2 and Co2p 1/2 were observed at around 779.8 eV and 795.6 eV, respectively, with an energy difference of approximately 15.8 eV between them, meanwhile, there were two satellite peaks around 786.2 eV and 801.0 eV; all these indicate the presence of Co^2+^ and Co^3+^ states. Figure 2c shows the XPS spectrum of element S, where the peak of S2p 3/2 at 163.9 eV and S2p 1/2 at 165.2 eV can be observed. The peaks observed in Figure 2d at 284.6 eV, 286.1 eV, and 288.6 eV correspond to C=C, C-N, and O-C=C, respectively, indicating the presence of carbon elements and their binding bonds in the composite material. Figure 2e displays two peaks of graphite N and pyridine N at 401.1 eV and 398.4 eV, respectively, which indicate the doping of the N element in the composite material. The full spectrum of the composite material confirms the presence of elements as Zn, Co, S, C, N, O. The extra peaks at around 532 and 228 eV represent O 1s and S 2s, respectively [30,31]. These tests confirm the successful synthesis of the ZnS/CoS@C/rGO composite material.

**Figure 3:**
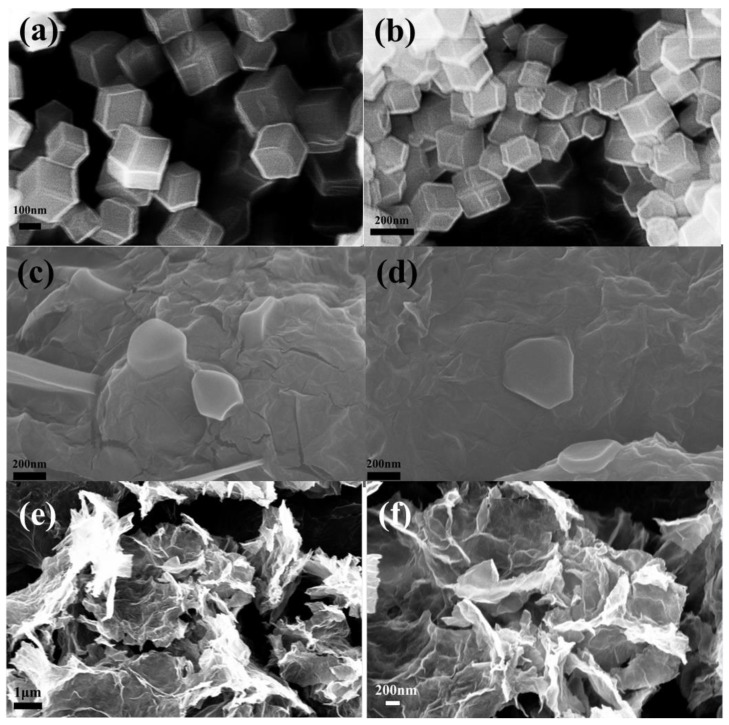
SEM image of (**a**,**b**) ZIF-8/67, (**c**,**d**) ZIF-8/67/rGO, (**e**,**f**) ZnS/CoS@C/rGO composite.

In Figure 3a,b, the structure of ZIF-8/67 before being combined with graphene is rhombic dodecahedral, and its particle size is around 200 nm. After the composite with graphene, the morphology of ZIF-8/67/rGO is as shown in Figure 3c,d and the ZIF-8/67 dodecahedron is embedded on the surface of graphene sheets. The excellent selectivity and stability of graphene-based materials largely compensate for the shortcomings of ZIFs. In other words, ZIF/graphene-based materials have a larger surface area and microporous volume, and they have more potential applications compared to the parent material. After vulcanization sintering, the final morphology of the ZnS/CoS@C/rGO composite is as shown in Figure 3e,f; the dodecahedron is mostly concealed by thick layers of graphene. Therefore, further analysis based on TEM results is needed. This results in a significant increase in the specific surface area of the composite material. Additionally, the active sodium storage sites in the composite material have also been increased, thereby augmenting the sodium storage capacity of ZnS/CoS@C/rGO.

**Figure 4:**
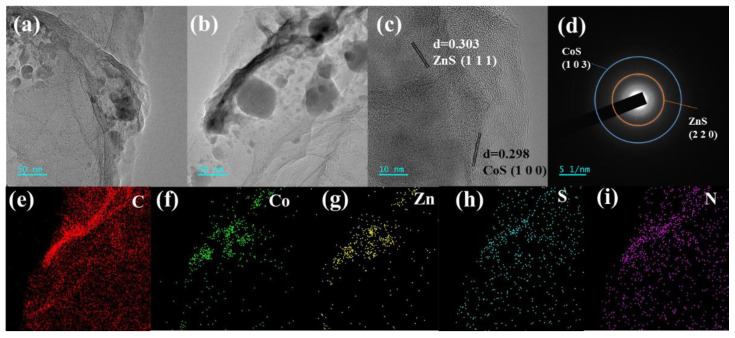
(**a**,**b**) TEM, (**c**) HRTEM, (**d**) the selected area electron diffraction (SAED) patterns of ZnS/CoS@C/rGO composite, (**e**–**i**) elemental mapping images of C, Co, Zn, S and N for the ZnS/CoS@C/rGO.

The TEM image (Figure 4a,b) shows that after high-temperature sintering, some parts of the ZIF-8/67 can still maintain a certain dodecahedral structure, but there is a phenomenon of structural collapse into a sphere, which is caused by the high-temperature sintering process. ZIF-8/67 is dispersed on ultra-thin graphene layers, which can effectively prevent the agglomeration phenomenon of ZIFs during the cycling process, thereby ensuring the cycling stability of the composite material. The HRTEM results show two crystal plane spacings of 0.298 and 0.303 nm, corresponding to the (1 0 0) and (1 1 1) crystal planes of CoS and ZnS, respectively. The selected area electron diffraction (SAED) patterns show two diffraction rings represent ZnS and CoS. The surface scanning results show that the composite material contains five elements of C, Co, Zn, S and N, which further proves the successful synthesis of the composite material.

**Figure 5:**
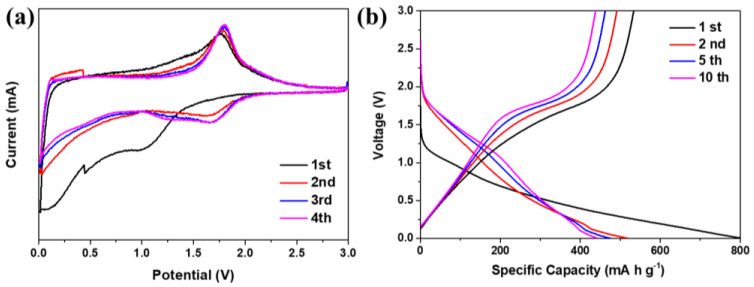
(**a**) CV curves under 0.1 mV s^−1^ for the first 4 cycles of the ZnS/CoS@C/rGO; (**b**) charge-discharge profiles of the ZnS/CoS@C/rGO under 200 mA g^−1^.

Figure 5a displays the cyclic voltammetry curve of the ZnS/CoS@C/rGO composite, measured at a scanning speed of 0.1 mV s^−1^. Upon the first cathode scan, a curve shift of approximately 1.2 V indicates the insertion of sodium ions into the ZnS/CoS lattice and the creation of SEI films. The reduction peak at 0.1 V corresponds to the conversion reaction of ZnS/CoS to Zn and Co. During the anodic process, the oxidation peak of around 1.7 V corresponds to the conversion of Zn/Co elements to ZnS/CoS, highlighting the reversibility of electrode reactions during electrochemical cycling. In subsequent cycles, the electrode reaction peak shifts slightly due to unavoidable polarization [32,33]. Figure 5b shows the 1st, 3rd, 5th, and 10th charge–discharge curves of ZnS/CoS@C/rGO composite cycling under 200 mA g^−1^. The potential plateau in these charge–discharge curves aligns with the voltage positions of the oxidation and reduction peaks that appear in the CV curve. During the initial discharge process, a turning point occurs at around 1.2 V, representing the first insertion of sodium ions and the formation of SEI films on the electrode surface. During the initial charging process, a clear plateau appears at around 1.7 V, representing the conversion process from Zn/Co to ZnS/CoS. The initial discharge and charging capacity of ZnS/CoS@C/rGO are 805.7 and 533.5 mAh g^−1^, respectively, and the ICE is calculated to be 66.2%. The low ICE may be due to the side reaction between the electrode and electrolyte, as well as the formation of the SEI film during the first cycle. However, the following cycle curve shows a Coulombic efficiency of nearly 100%.

**Figure 6:**
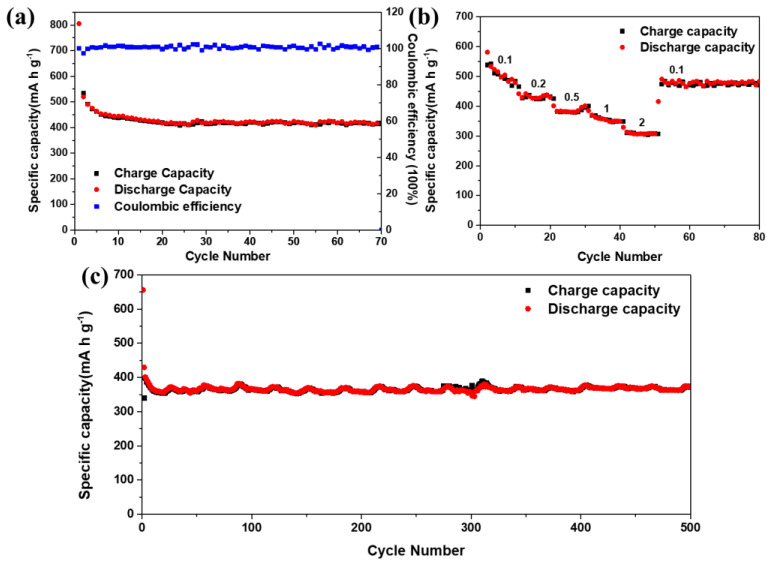
(**a**) Cyclic performance of ZnS/CoS@C/rGO composite under a current density of 200 mA g^−1^, (**b**) rate performance of ZnS/CoS@C/rGO composite under a different current density of 0.1, 0.2, 0.5, 1, 2 A g^−1^, (**c**) long cyclic performance under 1 A g^−1^.

The cycling performance of the ZnS/CoS@C/rGO composite material was tested in Figure 6a at a current density of 200 mA g^−1^. After 70 cycles, the composite material was able to maintain a capacity of 414.8 mAh g^−1^, indicating stable cycling performance without significant attenuation. This improvement is due to the increased specific surface area and improved morphology of sulfides derived from metal–organic frameworks combined with graphene. Figure 6b shows the rate performance of the composite material, with battery capacity remaining at 484.2, 432.2, 401.2, 349.0, and 308.0 mAh g^−1^ after 10 cycles at current densities of 0.1, 0.2, 0.5, 1, and 2 A g^−1^ respectively. When the current density was restored to 0.1 A g^−1^, the reversible capacity remained stable at 483.3 mAh g^−1^. Under gradually increasing current density, the reversible capacity did not experience significant attenuation, and the capacity remained the same after returning to a small current. This indicates that large currents do not have an excessive impact on the structure of the ZnS/CoS@C/rGO composite material. Figure 6c displays the long cyclic performance of the composite under a large current density of 1 A g^−1^. Even after 500 cycles, the ZnS/CoS@C/rGO composite material was able to maintain a capacity of 374.2 mAh g^−1^, proving its excellent cycle life under high current conditions.

**Figure 7:**
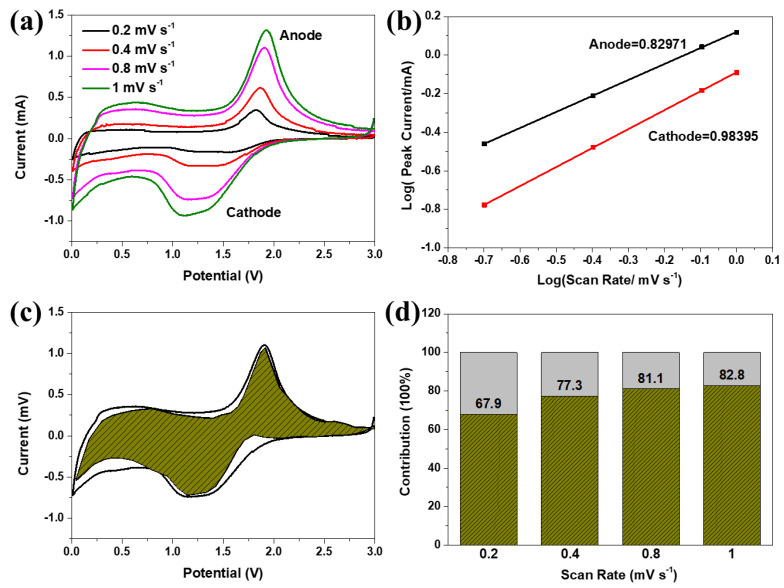
Electrochemical pseudocapacitance behavior of ZnS/CoS@C/rGO electrode: (**a**) CV curves measured in the range of 0.2–1.0 mV s^−1^, (**b**) the relationship between log (scan rate, mV s^−1^) and log (peak current, A), (**c**) at a scan rate of 0.8 mV s^−1^, 81.1% of the total capacity comes from the pseudocapacitance behavior; (**d**) the contribution ratio of pseudocapacitance at different scan rates.

In order to further study the electrochemical storage process characteristics of the ZnS/CoS@C/rGO electrode, CV curves were tested at different scanning rates ranging from 0.2 to 1 mV s^−1^ (Figure 7a). The relationship between log (scan rate, mV s^−1^) and log (peak current, A) can be expressed by the following equation.

i = av^b^         log(i) = blog(v) + log(a)

The parameters a and b are adjustable, and the behavior of the electrochemical reaction is dependent on the value of b. When the b value approaches 0.5, the ion diffusion controls the reaction, while the pseudocapacitor controls it when the b value is closer to 1 [34,35]. The graph in Figure 7b shows a linear relationship between log(i) and log(v). The anode and cathode have corresponding redox peaks with b values of 0.82971 and 0.98395, respectively. This indicates that the ZnS/CoS@C/rGO electrode’s redox process has partial pseudocapacitance behavior, resulting in excellent high-rate characteristics. The total current at a fixed potential is a combination of the pseudocapacitance mechanism (k_1_v) and ion diffusion process (k_2_v^1/2^), which can be calculated using the following formula:

i = k_1_v + k_2_v^1/2^

Here, k_1_v and k_2_v^1/2^ represent the pseudocapacitance process and ion diffusion process [36,37]. The proportion of pseudocapacitance at a scanning rate of 0.8 mV s^−1^ is shown in Figure 7c. The yellow shaded area represents the proportion of pseudo capacitor capacity, which is approximately 81.1%. Figure 7d shows that as the scanning rate increases from 0.2 to 1 mV s^−1^, the contribution rates of pseudocapacitance are 67.9%, 77.3%, 81.1%, and 82.8%, respectively. This indicates that as the scanning speed increases, the contribution of pseudocapacitance to the total capacitance also increases.

**Figure 8:**
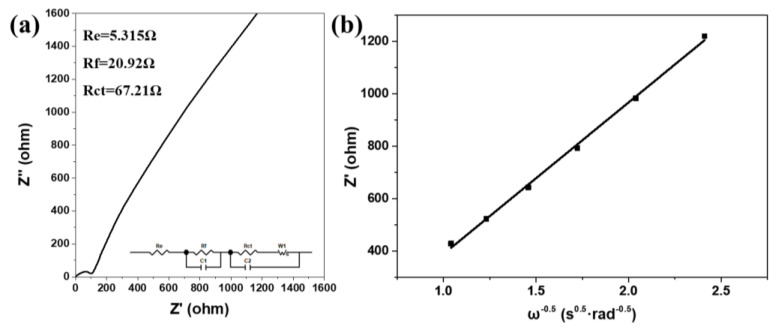
(**a**) EIS plots of ZnS/CoS@C/rGO after 70 cycles under 200 mA g^−1^, (**b**) Z’ vs. ω^−0.5^ plots calculated from EIS under low frequency.

The composite material’s electroconductibility and charge transfer speed improvement were evaluated after 70 cycles under a current density of 200 mA g^−1^ using EIS curves and fitting results. As seen in Figure 8a, the impedance curves of ZnS/CoS@C/rGO were made up of semicircles and inclined straight lines, indicating that the material’s electrochemical kinetics can be divided into two steps. Initially, sodium ions migrate through the electrolyte to reach the electrode surface’s vicinity, allowing the external circuit to transfer electrons to the electrode surface to maintain charge balance. During this period, the semicircle of EIS represents the formation of SEI layer impedance (Rf), charge transfer impedance (Rct), and double capacitance impedance (C). Then, a large amount of sodium ions accumulate on the electrode surface, resulting in a higher Na^+^ concentration on the electrode surface than internally. The resulting Na^+^ concentration gradient causes sodium ions to diffuse from the electrode surface to the interior to maintain balance, producing a diffusion impedance known as “Warburg impedance” (Ws), which is reflected in the slash part of the EIS results. Additionally, Re represents the battery’s impedance, which is reflected in the intercept of the high-frequency region Z′ [38]. The fitting results of ZnS/CoS@C/rGO electrodes’ Re, Rf, and Rct were 5.315, 20.92, and 67.21 Ω, respectively.

In addition, the fitting results in the low-frequency region can be used to calculate the diffusion coefficient of Na^+^ (D_Na_^+^). The calculation equations are as follows: [39,40]:

$$Z^{\prime }=R_{e}+R_{ct}+\sigma \omega^{-0.5}\;D_{{Na}^{+}}=\frac{{\left(RT\right)}^{2}}{{2(An^{2}F^{2}C\sigma )}^{2}}$$

In this context, certain letters and symbols represent scientific values and measurements. For example, the letter R stands for the ideal gas constant, which has a numerical value of 8.314. The letter T represents the absolute temperature in Kelvin, with a specific value of 298.15 K. The letter n is used to represent the number of electrons involved in the electrochemical process. The letter F, on the other hand, stands for the Faraday constant, which has a numerical value of 96,500 C mol^−1^. The letter C represents the concentration of sodium ions. Additionally, the symbol ω represents the angular frequency, while the symbol σ represents the Warburg factor, which can be calculated from Z′ fitting. A graph, as shown in Figure 8b, displays the relationship between Z′, ω, and 0.5. The sodium diffusion coefficient for the ZnS/CoS@C/rGO is determined to be 2.74 × 10^−10^ cm^2^ s^−1^. The performance comparison between ZnS/CoS@C/rGO composite material and similar materials in other studies is shown in Table 1.

## 3. Materials and Methods

### 3.1. Synthesis of ZIF-8/67 Dodecahedron

To create a ZIF-8/67 dodecahedron, start by dissolving 0.7 g Zn(NO_3_)_2_·6H_2_O and 0.7 g Co (NO_3_)_2_·6H_2_O in 100 mL of methanol. Stir the mixture well and label it as solution 1. Next, dissolve 3.8 g 2-methylimidazole and 0.8 g CTAB in 100 mL methanol and stir evenly to create solution 2. Under vigorous stirring, pour solution 1 into solution 2 quickly. After stirring the mixture for five minutes, let it sit at room temperature for 24 h. Centrifuge the mixture to obtain the product. Wash it with methanol three times and bake it in a vacuum oven at 150 °C for 8 h to obtain the desired ZIF-8/67 dodecahedron.

### 3.2. Synthesis of ZIF-8/67/GO

To create ZIF-8/67/GO, start by weighing 0.14 g ZIF-8/67 and mixing it with a specific amount of graphene solution that was prepared using the Hammers method. Adjust the concentration to 1 mg mL^−1^, add water until the mixture reaches 100 mL, and stir for 30 min until even. Next, add 1.44 g of L-ascorbic acid as the reducing agent and stir it at ultrasonic speed until there is no precipitation at the bottom. Heat the mixture in a water bath at 95 °C for 2 h to form a hydrogel, and then dry it using the freeze-drying method. This will result in the creation of ZIF-8/67/GO.

### 3.3. Synthesis of ZnS/CoS@C/rGO

Place sulfur powder in a tube furnace at a distance of 10–13 cm upstream of ZIF-8/67/GO (mass ratio of 4:5, usually 80 mg sulfur powder with 100 mg ZIF-8/67/GO), raise the temperature at a rate of 2 °C min^−1^ to 600 °C under nitrogen atmosphere, and hold for two hours to obtain the final product, which is recorded as ZnS/CoS@C/rGO.

### 3.4. Material Characterization

To analyze the crystal structure of the as prepared material, X-ray diffraction (XRD) was used, while X-ray photoelectron spectroscopy (XPS) was utilized for valence analysis of the elements. Raman testing was conducted to analyze the structure information of the carbon material. The JY Company’s instrument HR800 was used during the tests, and a laser wavelength of 532 nm was employed. The thermogravimetric test (TGA) was performed to determine the content of ZnS/CoS/C in the composite material, under an air atmosphere, within the temperature range of 25–800 °C. A scanning electron microscope (SEM) was used to analyze the morphology and element distribution of the composite material, while a transmission electron microscope (TEM) was used to observe its internal structure. The crystal structure of the material was analyzed further by high-resolution transmission electron microscopy (HRTEM) and selected area electron diffraction pattern (SAED).

### 3.5. Electrochemical Measurements

The electrochemical performance of the ZnS/CoS@C/rGO composite material was analyzed by assembling a coin battery with sodium as the counter electrode. We mixed the composite material with the conductive agent (Super-P) and binder (sodium alginate) in a ratio of 6:2:2 to form a uniform slurry. We applied the slurry on copper foil and dried it thoroughly in a vacuum at 120 °C for more than 10 h. We used 1 M sodium perchlorate with 5% FEC dissolved in 1:1 ethylene carbonate and diethyl carbonate as the electrolyte. We tested the cycle and rate performance using the Neware battery test system. We also conducted cyclic voltammetry (CV) using a different scan rate (0.1–1 mV s^−1^) with CS350.

## 4. Conclusions

In summary, a composite material made by combining graphene and metal–organic framework-derived sulfide was optimized to create the ZnS/CoS@C/rGO composite material, which was used as the anode material for sodium-ion batteries. This composite material has been shown through electrochemical tests to have good cycle performance. Even after 70 cycles, the capacity of 414.8 mAh g^−1^ can still be maintained at a current density of 200 mA g^−1^, which indicates that the improved morphology contributes to the improvement of cycle stability and cycle life. In addition, in the rate performance test, even if the current density increases to 2 A g^−1^, the reversible capacity can still maintain 308.0 mAh g^−1^. Part of the composite’s pseudocapacitive contribution is credited for its excellent rate performance. The composite of metal–organic framework-derived sulfides and graphene overcomes the volume change during charging and discharging. It is expected that the metal–organic framework-derived sulfide/graphene composite will play an important role in the application of anode materials for sodium-ion batteries.

## Figures and Tables

**Table 1:** Comparison of sodium storage properties of ZnS/CoS composite materials.

Materials	Current Density (A g^−1^)	Cycle Number	Specific Capacity (mAh g^−1^)	Ref.
H-ZnS@C	0.1	240	760	[41]
CoS⊂NSC	0.1	400	305.6	[42]
CoS_2_/NiS_2_-RGO	0.1	50	473.7	[43]
Sb/ZnS@C	0.1	150	554.8	[44]
ZnS/graphene composites	0.1	100	491	[45]
CoS@S-doped OLC	0.1	500	582	[46]
CoS@rGO@C	0.1	100	567	[47]
SSM/CoS	0.05	50	313	[48]
ZnS/GAs	0.1	100	539	[49]
CoS@BHCS	1	400	543	[50]
ZnS@NSC	1	100	552.6	[51]
0.7ZnS:0.3g-C_3_N_4_	1	750	432.6	[52]
(CoS NP@NHC)@MXene	2	650	420	[53]
ZnS/CuS@C	2	700	389.4	[54]
CC-ZnS/CNT	5	500	314	[55]
ZnS@CNFs	0.1	50	428.7	[56]
ZnS/N-CX	0.1	5	312	[57]
Cu2S@ZnS/C	10	1000	333	[58]
ZnS:SnS@CNT	0.1C	100	364	[59]
(CoSn)S/C	2	1000	368	[60]
CoS/NSCNF	6	2250	358.6	[30]
ZnS/Sn_2_S_3_@C	0.5	100	413	[61]
ZnS/CoS@C/rGO	0.2	70	414.8	This work
	1	500	374.2	

## Data Availability

The data presented in this study are available upon request from the corresponding author.

## Sample Availability

Samples of the compounds ZnS/CoS@C/rGO are available from the authors.
